# Cactus Grandiflora

**Published:** 1891-07-11

**Authors:** 


### THERAPEUTICS.

### CACTUS GRANDIFLORA.

The Cactus grandiflora, or night-blooming cereus, is a well-known flowering plant, and has of late been used in medicine, though by no means to the extent it would seem to deserve, judging from our own favourable experience and the few reports of others.

Its sphere of action appears to be limited to diseases and affections of the heart. In functional palpitation associated with dyspepsia we have found the tincture in one-drop doses every quarter of an hour of the greatest service, the first dose generally sufficing to relieve. It does not invariably give such happy results, but a trial of this remedy in about a hundred cases during the last three years enables us to state confidently that, in suitable cases, we have found it surpasses any of the better known drugs used for such cases. The patients usually complain of violent palpitation coming on half an hour or an hour after meals, and more especially, too, when lying in bed at night. The palpitation is not observed, as a rule, during exertion. When there is catarrh of the stomach the addition of two drops of the tincture to a bismuth mixture, administered three times a day, will often obviate the necessity for its administration separately. We recently had occasion to prescribe for a female dyspeptic who suffered intensely from palpitation and great nervousness. All the usual remedies had been tried without much relief by her medical attendant. The simple addition of two minims of cactus to her bismuth mixture cured the palpitation and the distressing sense of fear associated with it. Dr. Watson Williams has found it useful in the paroxysmal tachycardia of certain neurotic subjects. The beneficial effects observed in such cases led him to employ the remedy in Grave's disease. In none of his cases was there any marked enlargement of the thyroid gland, but the greatly increased frequency of the pulse, the presence of exophthalmos, Von Graefe's symptom, and other minor symptoms left no donbt as to the nature of these cases. The cactus may, in some cases, be advantageously combined with iron and arsenic.

In organic affections of the heart it generally disappoints, and is certainly inferior to digitalis, strophanthus, and many other familiar cardiac stimulants. It is an old popular remedy in certain districts, for cardiac dropsies, and it does tend to help them. We found the urine increased in amount from its use, and in one case the improvement in the character of the pulse as well as the increase in amount of urine passed was about the same when five drops of cactus was administered every four hours, as when digitalis was given in ten minim doses three times a day, in each case the tincture being given for several days in succession. The smaller doses referred to in the earlier part of this article for functional palpitation are useless in organic diseases of the heart, for which thirty to forty-five minims daily must be administered.

